# Ulcer like projection: a case report and follow up

**DOI:** 10.11604/pamj.2016.24.151.9781

**Published:** 2016-06-21

**Authors:** Zairi Ihsen, Khadija Mzoughi, Jnifene Zouhaier, Ben Moussa Fathia, Sonia Segaier, Sofiene Kamoun, Sana Fennira, Sondos Kraiem

**Affiliations:** 1 Department of Cardiology, Habib Thameur Hospital, Bab El Fallah, 2004, Tunis, Tunisia; 2 Department of Radiology, Habib Thameur Hospital, Bab El Fallah, 2004, Tunis, Tunisia

**Keywords:** Aorta, surgery, aortic intramural hematoma, tomography, transesophageal

## Abstract

Here we report the case of a 57-year-old female with an aortic intramural hematoma that was treated with medical approach. Follow confirmed the favorable clinical evolution.

## Introduction

The definition of ulcer like projections (ULP) remains unclear. Some studies have shown that the presence of ULP is intramural aortic hematoma (IMH) is associated with a progressive disease course.

## Patient and observation

**Figure 1:**
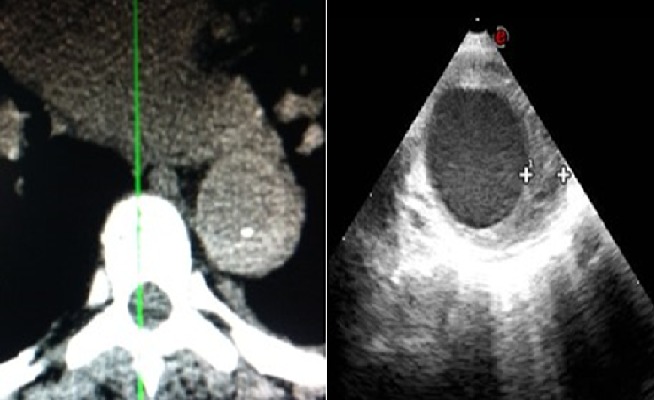
Type B hematoma formation in the descending thoracic aorta with internal deviation of intimal calcification. At left: non-contrast computed tomography. At right: TEE showing tickening of aorta

**Figure 2:**
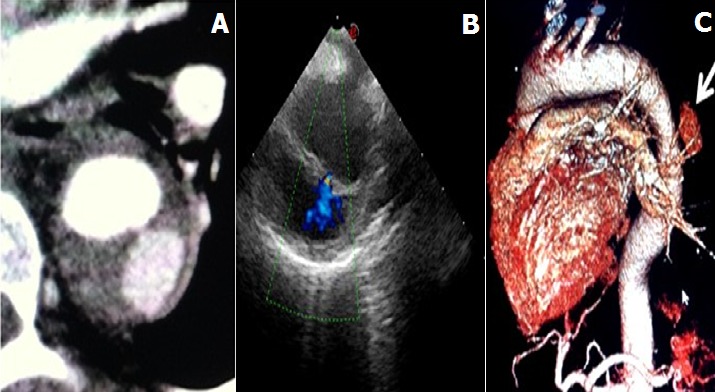
Intimal tear, with a reperfusion area in the hematoma. A) contrast enhanced CT scan; B) TEE; C) CT scan with reconstruction

**Figure 3:**
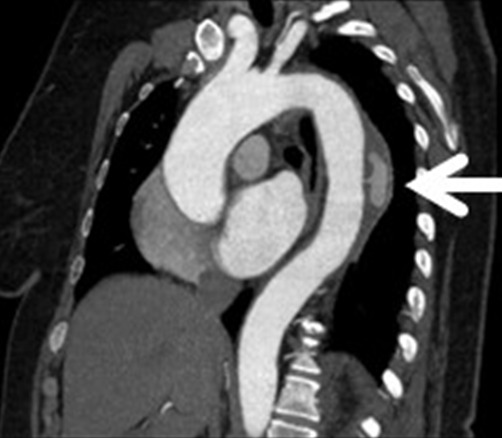
Enhaced CT scan 3 months later: ectasic aorta with stable hematoma

A 57-year-old female, with a history of severe hypertension presented with interscapular pain with a sudden onset. On physical examination, she had a high blood pressure 220/120. On electrocardiogram there was a sinus rhythm with a left ventricular hypertrophy. Initial non-contrast CT and transoesophagel echography (TEE) (Figure 1) showed a Stanford type B hematoma formation in the descending thoracic aorta with internal deviation of intimal calcification. Two weeks later, after blood pressure stabilization, a control CT scan and TEE (Figure 2 A, B) revealed ULP, appearing as an intimal tear, with a reperfusion area in the hematoma. Contrast CT scan showed the importance of the reperfusion area (Figure 2 C). On the 3 months follow up contrast CT scan (Figure 3), there was no aortic dissection or aneurysm. The hematoma was stable with an ectasic aorta.

## Discussion

Aortic intramural haematoma (IMH) is an entity included in the acute aortic syndrome. corresponds to blood collection within the media, resulting in a cleavage of the intima [1]. Historically, two mechanisms have been described to explain the formation of the hematoma: Spontaneous rupture of the vasa vasorum vessels in the aortic wall leading to weakening of the intima and resulting in a clivage wall, and athero-sclerotic plaque rupture resulting in penetration of blood into the media layer. in CT, it's described as a localized or circumferential thickening of the aortic wall, spontaneously hyper dense, not enhanced after contrast injection. In TEE, it appears as a low thickening of the aortic wall. The description of the aortic hematoma in imaging, excluded any intimo-medial rupture. The proof of the existence of these lesions was made by surgeons who describe the presence of intimo-medial ruptures causing the hematoma with anterograde or retrograde extension of rupture. Advances in imaging have enabled the detection of smaller lesions [2]. Emergency treatement is based on analgesic treatment, control blood pressure <120 mmHg and heart rate <60 btm. Endovascular treatment is indicated if there are signs of malperfusion. On the acute phase, in case of complicated hematoma, intimal tear is treated with covered stent. Their exclusion allows the healing of hematoma (including area not covered). This type of treatment is also possible for Type A hematoma when the intimal tear is open and that surgery is not desirable (comorbidities, traumatic context) [3]. With monitoring, these lesions can continue to evolve regardless of whether regression of wall thickening. If aneurysm appears, treatment with stent graft can be considered. CT monitoring is required, even in case of favorable clinical evolution. In contrast to type A IMH, type B IMH appears to have a slightly more benign prognosis than type B [4]. type B IMH patients compose the group in which regression is most often seen [5]. These lesions are often grouped under the term “ULP” (like Ulcer Projection) in the Anglo-Saxon literature. In our case, at short time follow up there were no complications associated to the presence of ULP.

## Conclusion

Aortic intramural haematoma, a variant form of classic aortic dissection, Modern imaging modalities enables to investigate the natural history and distinctive clinical features of this disease entity. Thus, careful follow up imaging study during the acute phase is absolutely necessary, especially with medical treatment in order to detect complications.
